# Magnetite nanoparticle decorated reduced graphene oxide for adsorptive removal of crystal violet and antifungal activities

**DOI:** 10.1039/d0ra07061k

**Published:** 2020-09-21

**Authors:** Mebrahtu Hagos Kahsay, Neway Belachew, Aschalew Tadesse, K. Basavaiah

**Affiliations:** Department of Chemistry, Woldia University P.O. BOX 400 Woldia Ethiopia hagosmebrahtu@gmail.com mebrahtuh@wldu.edu.et; Department of Chemistry, Debre Berhan University P.O. BOX 445 Debre Berhan Ethiopia; Department of Applied Chemistry, Adama Science and Technology University P.O. BOX 1888 Adama Ethiopia; Department of Inorganic and Analytical Chemistry, Andhra University Visakhapatnam 530003 India

## Abstract

This work reports the synthesis and application of magnetic rGO/Fe_3_O_4_ NCs using a pod extract of *Dolichos lablab* L. as areducing agent. GO was synthesized by a modified Hummers method, however GO was reduced using the plant extract to produce rGO. The as-synthesized rGO/Fe_3_O_4_ NCs were characterized by UV-vis spectrophotometer, Fourier transform infrared (FT-IR) spectroscopy, FT-Raman spectroscopy, X-ray diffraction (XRD), field emission scanning electron microscopy supported with energy dispersed X-ray spectroscopy (FESEM-EDX), transmission electron microscopy (TEM) and vibrating sample magnetometer (VSM). The synthesis of magnetic rGO/Fe_3_O_4_ NCs was confirmed from characterization results of FT-Raman, TEM and VSM. The FT-Raman results showed the D and G bands at 1306.92 cm^−1^ and 1591 cm^−1^ due to rGO and a peak at around 589 cm^−1^ due to Fe_3_O_4_ NPs that were anchored on rGO sheets; TEM results showed the synthesis of Fe_3_O_4_ with an average particle size of 8.86 nm anchored on the surface of rGO sheets. The VSM result confirmed the superparamagnetic properties of the rGO/Fe_3_O_4_ NCs with a saturation magnetization of 42 emu g^−1^. The adsorption capacity of rGO/Fe_3_O_4_ NCs towards crystal violet (CV) dye was calculated to be 62 mg g^−1^. The dye removal behavior fitted well with the Freundlich isotherm and the pseudo-second-order kinetic model implies possible chemisorption. Besides, rGO/Fe_3_O_4_ NCs showed antifungal activities against *Trichophyton mentagrophytes* and *Candida albicans* by agar-well diffusion method with a zone inhibition of 24 mm and 21 mm, respectively. Therefore, rGO/Fe_3_O_4_ NCs can be used as an excellent adsorbent to remove organic dye pollutants and kill pathogens.

## Abstract 2

This work reports synthesis of magnetic rGO/Fe_3_O_4_ NCs using pod extract of *Dolichos lablab* L. as a reducing agent and its applications.

## Graphical abstract

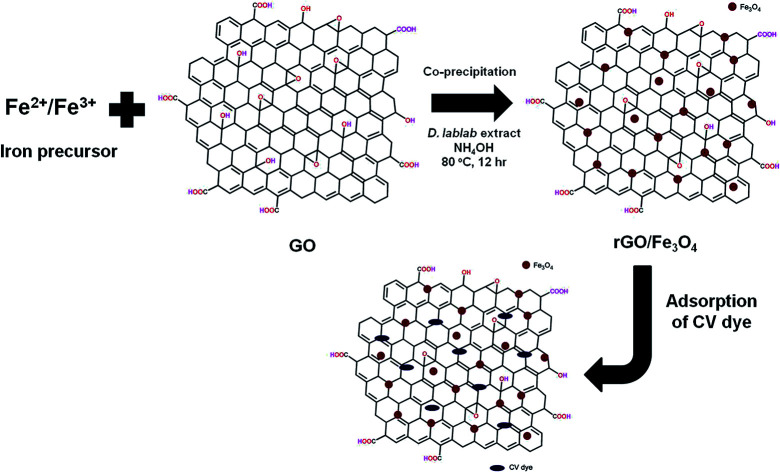

## 1. Introduction

Graphite oxide, also known as graphitic oxide or graphitic acid, is a compound of carbon, oxygen and hydrogen in variable ratios, obtained by treating graphite with strong oxidizers.^1^ Most procedures have been reliant on strong oxidizing mixtures containing one or more concentrated acids and oxidizing materials during the synthesis of graphitic oxide.^2,3^ The bulk material disperses in basic medium to yield monomolecular sheets or one atom thick in a closely packed honeycomb two dimensional (2D) lattice known as GO.^4^ Hence, graphene is the single-layer form of graphite. The combination of GO or rGO with magnetite produces Fe_3_O_4_–graphene hybrid which has a planar geometry, high conductivity, fascinating carrier transport properties, large surface area, strong magnetism, low cost and environmentally benign nature which has opened an opportunity for various applications.^5–8^ Therefore, rGO/Fe_3_O_4_ hybrids have reported for catalysis, water purification, sensors, toxic heavy metal removal, capacitors, bio-medical diagnosis therapy, microwave absorption, water desalination, photocatalysis and antimicrobial.^7,9–15^ The hybrid combination of magnetic Fe_3_O_4_ NPs and graphene sheets best produces rGO/Fe_3_O_4_ NCs that have recognized properties such as great dispersibility, large surface area, superparamagnetism and fabulous extraction capacity.^16^ The π–π stacking, hydrogen bonding and electrostatic interaction between rGO/Fe_3_O_4_ NCs and dye molecules are responsible for the adsorptive removal of dye molecules from aqueous solution.^17^ Moreover, preceding reports indicated that rGO/Fe_3_O_4_ NCs can efficiently remove toxic heavy metals,^7,18^ fluoroquinolones,^19^ organic dyes^16,20^ and MB dye *via* degradation.^17^

In general, centrifugation and filtration methods are used to separate the adsorbent material from aqueous solution^21^ even though these applications are time-consuming and require extra cost.^22^ Compared with traditional centrifugation and filtration methods, magnetic separation method is an efficient, fast and economic method for the separation of magnetic adsorbents from the medium after the adsorption treatment of pollutants is completed.^23^ Hence, it is important to synthesize magnetically separable and high surface area rGO/Fe_3_O_4_ NCs for removal of water pollutants and treat pathogens. Diverse methods have been used to synthesize rGO/Fe_3_O_4_ NCs, such as chemical co-precipitation,^13,14^ solvothermal reduction,^11,24^ chemical reduction,^7^ and green synthesis.^12,15,25^ Different types of adsorbents have been used as way of removal of organic compounds and metals from aqueous solution. For example, Harijan and Chandra^26^ have stated facile fabrication of magnetite graphene composite through thermal reduction of graphene oxide to graphene in alkali medium with extreme sorption capacity for Cr^6+^ (5.5 mg g^−1^) at pH = 6.6. The graphene sheets prohibited agglomeration of the Fe_3_O_4_ NPs while enabling a good dispersion of the Fe_3_O_4_ NPs and at the same time the specific surface area of the composite is considerably enhanced. Similarly, Sun *et al.*^11^ have reported one step solvothermal synthetic route of rGO/Fe_3_O_4_ NCs for excellent removal of toxic dyes, *i.e.*, rhodamine B and malachite green with removal efficiency of 91% and 94%, respectively. He *et al.* have reported waste biofilms biosorbents to treat Cd^2+^ from aqueous solutions. The maximum adsorption capacity of dry waste biofilms for Cd^2+^ is 42 mg g^−1^ when the initial concentration of Cd^2+^ is 50 mg L^−1^.^27^ Bionanomaterials such as *Saccharomyces cerevisiae* and nano Fe_3_O_4_ encapsulated in a sodium alginate–polyvinyl alcohol matrix and *Penicillium* doped with nano Fe_3_O_4_ entrapped in polyvinyl alcohol–sodium alginate gel beads have been reported to remove atrazine from aqueous solution by biodegradation.^28,29^ Moreover, synthesis of rGO/Fe_3_O_4_ NCs through a facile and environmental benign approach is a prime concern for practical application. Plant extracts currently attract a tremendous research interest for synthesis of rGO/Fe_3_O_4_ NCs owing to environmentally friendly and ability to reduce GO into rGO. Gurunathan *et al.*^30^ have recorded reduction of GO into rGO with spinach leaf extract as a simultaneous reducing and stabilizing agent. The plant extracts of *Solanum trilobatum*^17^ and *Averrhoa carambola*^12^ were previously reported as reducing agent for synthesis of rGO/Fe_3_O_4_ NCs. To the best of our information, *Dolichos lablab* L. has not been reported to synthesize rGO or rGO/Fe_3_O_4_ NCs. *Dolichos lablab* L. is a plant that is extensively scattered in India that contains lectins sugar in the form of mannose/glucose specific,^31^ galactose specific,^32^ crude lipid, crude protein, insoluble dietary fibre, soluble dietary fibre, carbohydrate, and amino acids.^33^ For this reason, the existence of many alternative phytoconstituents of the plant motivated the researchers to prepare rGO/Fe_3_O_4_ NCs for different applications.

Herein, in this study, the researchers have synthesized rGO/Fe_3_O_4_ NCs using a facile co-precipitation method of Fe_3_O_4_ onto rGO sheet in the presence of extract of *Dolichos lablab* L. for the adsorptive removal of crystal violet (CV) and antifungal activity against pathogens. The pod extract of *Dolichos lablab* L. was used as reducing and capping agent during preparation of rGO and rGO/Fe_3_O_4_ NCs. The crystal structure, electronic property and surface morphology of the as-synthesized materials were investigated by UV-vis, FT-IR, FT-Raman, powder XRD, FESEM-EDX, TEM and VSM techniques. The CV dye was selected as a model water pollutant to investigate the adsorption capacity of rGO/Fe_3_O_4_ NCs. Besides, *Trichophyton mentagrophytes* and *Candida albicans* pathogens were chosen to examine the antifungal activities.

## 2. Experimental

### 2.1. Materials

All the chemicals in this paper were analytical grade acquired from Merck, HiMedia and Sigma-Aldrich and used without further purification. Iron(ii) sulfate heptahydrate (FeSO_4_·7H_2_O), hydrogen peroxide (H_2_O_2_, 30% wt), sodium nitrate (NaNO_3_, 98%), concentrated sulfuric acid (H_2_SO_4_, 98% wt), potassium permanganate (KMnO_4_), crystal violet dye (C_25_H_30_ClN_3_) and sodium hydroxide (NaOH) were from Merck, India; iron(iii) chloride hexahydrate (FeCl_3_·6H_2_O) from HiMedia, India; and Graphite flake with +100 mesh from Sigma-Aldrich, India. The standard strains of *Trichophyton mentagrophytes* and *Candida albicans* (fungus) were obtained from Adhya Biosciences Pvt. Ltd., Visakhapatnam. Milli-Q water was used in the experiment. Pocket-sized pH meter acquired from Hanna instruments was used to regulate the pH of the solutions. Hot Air Oven, Kemi was used to dry washed samples. *Dolichos lablab* L. pods were collected from market near Andhra University, Vishakhapatnam, India.

### 2.2. Preparation of aqueous pod extract of *Dolichos lablab* L

Pod layers were first detached from the seeds and cut into pieces by hand and shade dried in the laboratory for 21 days. Dried pods were grinded into powder using the Bajaj (Gx8) mixer grinder. The optimization of percent aqueous plant extract was taken based on our previous work.^34^ To prepare 1% pod extract of *Dolichos lablab* L., 1 g pod powder was poured into 250 mL Erlenmeyer flask containing 100 mL Milli-Q water and heated at 70 °C for 20 min. The solution broth was permitted to cool and later filtered with Whatman no. 42 filter paper to create yellowish solution. Lastly, the plant extract suspension was preserved in a refrigerator at 4 °C for further utilize.

### 2.3. Synthesis of graphene oxide and reduced graphene oxide

GO was processed by modified Hummers strategy.^1^ To prepare rGO from GO, pod extract of *Dolichos lablab* L. was used as a reducing agent. 100 mg of GO was added into round bottomed flask containing 50 mL Milli-Q water and sonicated for 30 min. Meanwhile 10 mL of 1% pod extract of *Dolichos lablab* L. was added and refluxed at 80 °C while stirring for 12 h to produce a black precipitate. Finally, rGO was collected by centrifugation at 12 000 rpm and dried at 100 °C in vacuum oven.

### 2.4. Synthesis of rGO-magnetite NCs using pod extract of *Dolichos lablab* L

The rGO/Fe_3_O_4_ NCs were fabricated through co-precipitation using FeSO_4_·7H_2_O and FeCl_3_·6H_2_O in the presence of GO in alkaline medium according to the procedure in ref. 35. One (1) mg mL^−1^ dispersed GO was exfoliated into 100 mL of iron source solution containing both FeSO_4_·7H_2_O and FeCl_3_·6H_2_O salts (1 : 2 molar ratio) and it was sonicated for 1 h. Ten (10) mL of 1% pod extract of *Dolichos lablab* L. solution was added slowly to the above suspension of GO at 30 °C with vigorous stirring under nitrogen atmosphere for 1 h. In short period, 10 mL of ammonia solution was added drop wise to modify the mixture pH to a value of 10. Moreover, reducing agents in the plant extract may reduce Fe^3+^ as well. Thereupon, the mixture was heated to 80 °C for 12 h to form dark colored solution indicating formation of rGO/Fe_3_O_4_ NCs. Finally, black colored rGO/Fe_3_O_4_ NCs was produced, separated, washed with Milli-Q water and ethanol, and then dried in hot air oven at 60 °C for 12 h.

### 2.5. Characterization

The UV-visible absorption spectra were recorded using a (UNICAM UV 500, Thermo Electron Corporation) spectrophotometer in the range of 200–800 nm. FT-IR spectrum was recorded over the range of 4000–400 cm^−1^ using a SHIMADZU-IR PRESTIGE-2 spectrometer. Raman spectrum was obtained using FT-Raman spectroscopy (Bruker RFS 27, USA) with laser source Nd (YAG 1064 nm) at 2 cm^−1^ resolution. 16+ mW laser power was irradiated on rGO/Fe_3_O_4_ NCs to collect Raman spectrum over the wide range 4000–50 cm^−1^. XRD patterns were recorded by PANalytical X'pert pro diffractometer at 0.02 degree per sec scan rate using Cu Kα_1_ radiation (*λ* = 1.5406 Å, 45 kV, 40 mA). TEM images were acquired through (TEM model FEI TECNAI G2 S-Twin) at an accelerating voltage of 200 kV. The morphology of the sample was characterized using FE-SEM (FE-SEM, Zeiss Ultra-60) equipped with EDX. VSM was used to examine the magnetic property of rGO/Fe_3_O_4_ NCs (Lakeshore Cryotronics, Inc., Idea-VSM, model 7410, USA).

### 2.6. Batch mode adsorption studies

The adsorption experiments of CV dye on rGO/Fe_3_O_4_ NCs took place with different initial concentrations of CV dye at different pH values at room temperature. 100 mL (5, 10, 15, 20 mg L^−1^) of CV dye solution was placed into 250 mL beaker and the pH (4–12) of the solution was maintained by using 0.1 M HCl/0.1 M NaOH solution. Afterward, 20 mg rGO/Fe_3_O_4_ NCs was added to the beaker and kept agitated in an incubator for 220 min until equilibrium was established. The initial concentration of the dye was reported as Co and in the consecutive 20 min time interval, concentration of the dye was recorded as *C*_e_. The amount of CV dye adsorbed per gram of adsorbent at time, *t*, *q*_*t*_ (mg g^−1^) and equilibrium, *q*_e_ (mg g^−1^) were determined using (eqn (1) and (2)).1
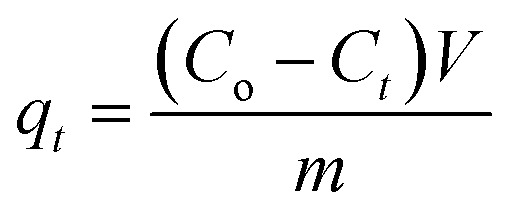
2
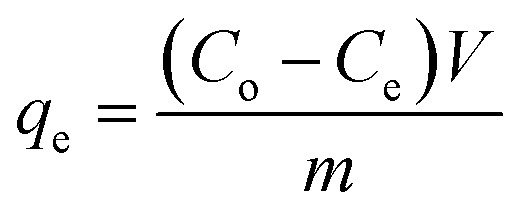


In addition, CV dye removal efficiency was calculated using (eqn (3)).3
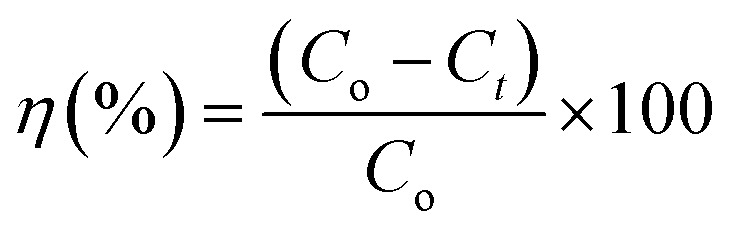
where, ‘*C*_o_’ is the initial CV dye concentration (mg L^−1^), ‘*C*_*t*_’ is concentration of CV dye at time, *t* (mg L^−1^), ‘*C*_e_’ is concentration of CV dye at equilibrium (mg L^−1^), ‘*V*’ is the volume of solution (L), ‘*m*’ is the mass of rGO/Fe_3_O_4_ NCs adsorbent (g) and ‘*η*’ is the dye removal percent (%). The adsorption isotherm was studied using Langmuir and Freundlich isotherm models, while, the kinetics was studied using pseudo-first and pseudo-second-order kinetic models.

### 2.7. Adsorption isotherms

To determine the maximum adsorption capacity of rGO/Fe_3_O_4_ NCs, the adsorption isotherm was analyzed by Langmuir^36^ and Freundlich^37^ isotherm models. The Langmuir adsorption isotherm argues consistent adsorbent surface activity and unilayer adsorption, with partial adsorbent active sites, is revealed as in (eqn (4)).4
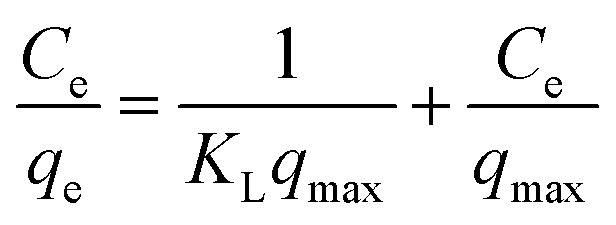
where, ‘*C*_e_’ is the equilibrium concentration of CV in the aqueous solution (mg L^−1^), ‘*K*_L_’ is the Langmuir adsorption constant (L mg^−1^) correlated to heat of adsorption, ‘*q*_max_’ is the maximal adsorption capacity of the adsorbent (mg g^−1^), and ‘*q*_e_’ is the amount of CV adsorbed per mass of adsorbent at equilibrium (mg g^−1^).

The Freundlich isotherm signifies surface heterogeneity of the adsorbent as well as multilayer coverage on the surface. The linear equation of Freundlich isotherm is expressed as in (eqn (5)).5
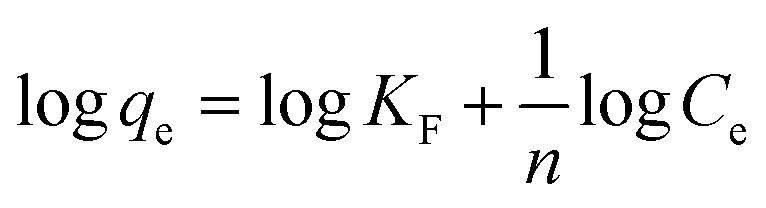
where, ‘*q*_e_’ is the quantity of dye adsorbed per unit weight of adsorbent (mg g^−1^), ‘*C*_e_’ is the equilibrium concentration of dye in the bulk solution (mg L^−1^), ‘*K*_F_’ is the Freundlich constant indicative of the relative adsorption capacity of the adsorbent (mg g^−1^) (L mg^−1^)^1/*n*^ and ‘*n*’ is adsorption intensity in the Freundlich equation.

### 2.8. Adsorption kinetics

The physical and chemical properties of the adsorbent as well as mass transfer mechanisms are among the several prominent parameters to determine the adsorption mechanism. To revise the effect of adsorption time on CV dye removal by rGO/Fe_3_O_4_ NCs, the mechanism of the adsorption process was studied by fitting pseudo-first-order and pseudo-second-order reactions to the experimental data. The adsorption equilibrium time of CV by rGO/Fe_3_O_4_ NCs was 200 min. The Lagergren pseudo first-order kinetic model^38^ is known as in (eqn (6)).

6 ln(*q*_e_ − *q*_*t*_) = ln *q*_e_ − *k*_1_*t*

where, ‘*q*_e_’ and ‘*q*_*t*_’ are amount of CV dye adsorbed at equilibrium and time, *t* (mg g^−1^), ‘*k*_1_’ is rate constant of pseudo-first-order kinetic model (min) and ‘*t*’ is time (min). Likewise, the pseudo-second-order kinetic model^39^ is expressed as in (eqn (7)).7
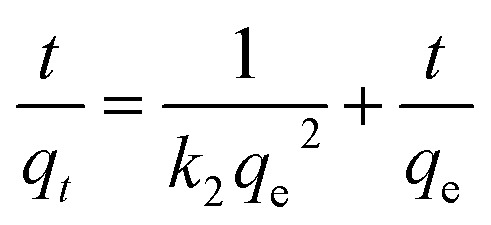
where, ‘*k*_2_’ is the rate constant of pseudo-second-order (g mg^−1^ min^−1^), ‘*q*_*t*_’ is the amount of CV dye adsorbed on surface of rGO/Fe_3_O_4_ NCs at time, *t* (mg g^−1^), ‘*q*_e_’ is the equilibrium sorption capacity (mg g^−1^).

### 2.9. Antifungal activity against *Trichophyton mentagrophytes* and *Candida albicans*

The antifungal activity was carried out using agar-well diffusion method by employing 24 h cultures with given rGO/Fe_3_O_4_ NCs.^40^ The medium was sterilized by autoclaving at 120 °C (15 lb per in^2^). About 20 mL of the nutrient agar medium/potato dextrose agar seeded with the respective fungal strains were transferred aseptically into each sterilized Petri plate. The plates were left at room temperature for solidification. Each plate, a single well of 6 mm diameter was made using a sterile borer. The rGO/Fe_3_O_4_ NCs were freshly reconstituted with suitable solvent (DMSO) and tested at various concentrations (2.5 mg mL^−1^, 5 mg mL^−1^, 10 mg mL^−1^). The samples (50 μL) and the control along with standard (clotrimazole (5 μg mL^−1^)) were placed in 6 mm diameter well. Then the fungal plates were incubated at 28 ± 2 °C. Activity diameter of the zone of inhibition was measured using Himedia antibiotic zone scale.

## 3. Results and discussion

### 3.1. Characterizations

#### 3.1.1. UV-vis analysis

**Fig. 1:**
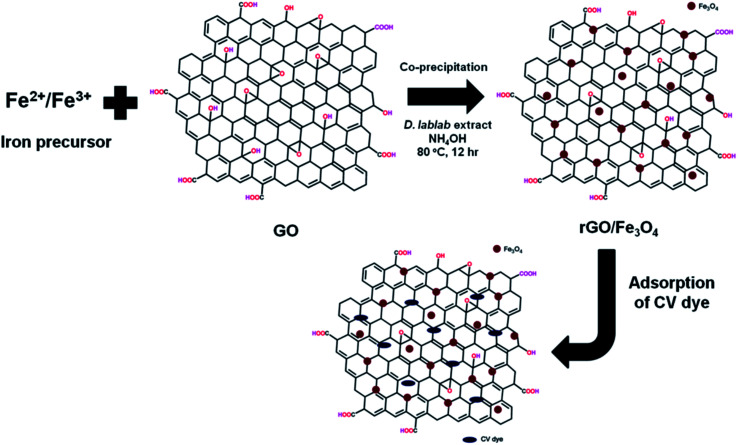
Proposed mechanism of synthesis of rGO/Fe_3_O_4_ NCs using *Dolichos lablab* L. extract and removal of crystal violet dye by adsorption.

**Fig. 2:**
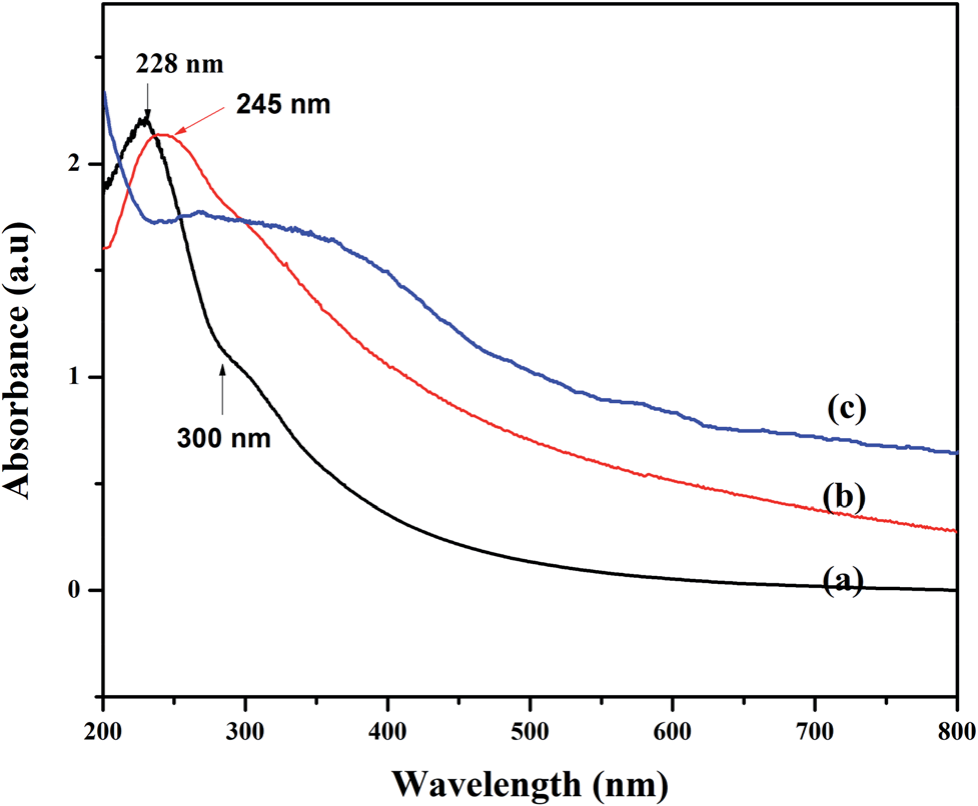
UV-vis spectra of (a) GO, (b) rGO and (c) rGO/Fe_3_O_4_ NCs.

In this study, one pot co-precipitation green method was used to synthesize rGO/Fe_3_O_4_ NCs using iron salts (Fe^3+^/Fe^2+^) in 2 : 1 molar ratio, GO and pod extract of *Dolichos lablab* L. A systematic schematic representation of synthesis of rGO/Fe_3_O_4_ NCs for the possible application of removal of CV dye is represented in Fig. 1. Fig. 2 depicts the absorption peak of the synthesized GO, rGO and rGO/Fe_3_O_4_ NCs by using UV-vis spectrophotometer. The absorption peak of GO at 228 nm is due to π to π* transition of the aromatic C=C bond,^41^Fig. 2(a). The reduction of GO using plant extract red shifted the peak to 245 nm is possible indication of synthesis of rGO, Fig. 2(b). Furthermore, a peak is observed for rGO/Fe_3_O_4_ NCs at around 267 nm, Fig. 2(c).

#### 3.1.2. FT-IR analysis

**Fig. 3:**
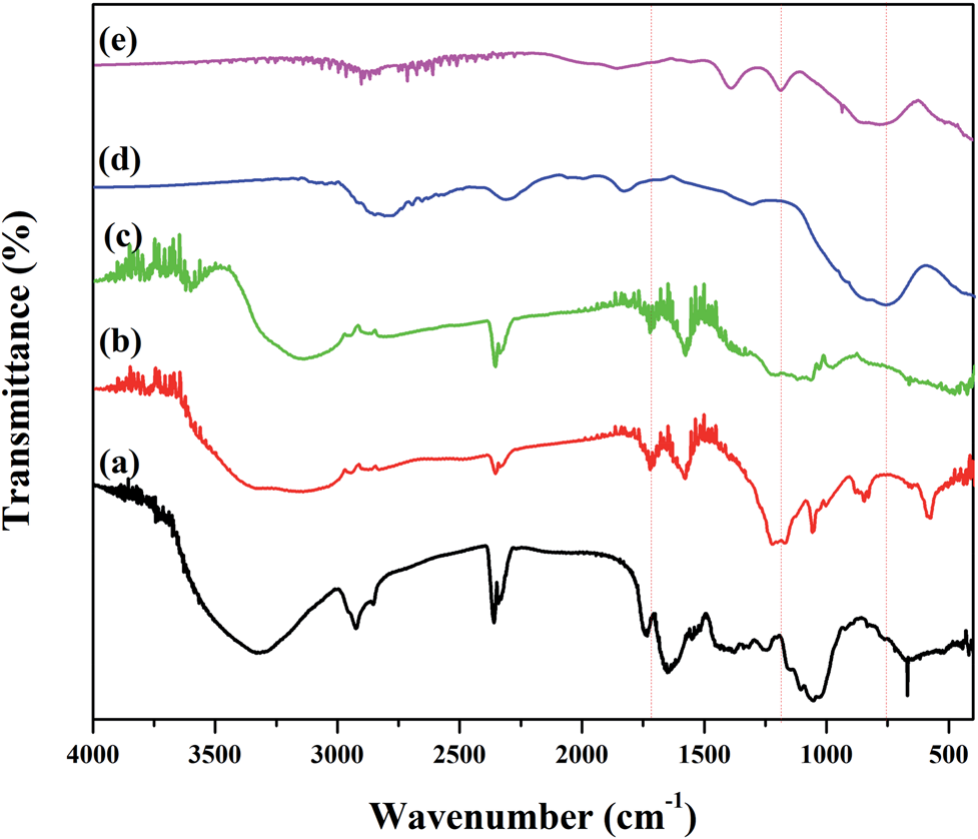
FT-IR spectrum of (a) pod extract of *Dolichos lablab* L., (b) GO, (c) rGO, (d) Fe_3_O_4_ NPs and (e) rGO/Fe_3_O_4_ NCs.

Fig. 3 depicts FT-IR spectrum to identify the functional groups of the plant extract, GO, rGO, Fe_3_O_4_ NPs and rGO/Fe_3_O_4_ NCs. Pod extract of *Dolichos lablab* L. showed absorption peaks at 3324, 2925, 1645, 1051 and 660 cm^−1^ due to O–H stretching, sp^3^ C–H stretching, C=O of amide or C=C stretching, C–O stretching and C–H bending, respectively (Fig. 3(a)). GO showed characteristic peaks at 3280 cm^−1^, 1702 cm^−1^ and 1194 cm^−1^ due to O–H, C=O and epoxy functional groups, respectively,^42^Fig. 3(b). The peak intensity 1194 cm^−1^ is very much decreased in the rGO spectrum implies pod extract of *Dolichos lablab* L. is excellent reducing agent. The O–H stretching peaks in Fig. 3(a–c) were disappeared in the spectrum of both Fe_3_O_4_ NPs and rGO/Fe_3_O_4_ NCs are strong indications that the plant extract with carboxylic functional group was used as reducing agent. In addition, FT-IR spectrum of rGO/Fe_3_O_4_ NCs showed characteristic peaks at 1184 and 820 cm^−1^ due to C–O stretching and CH_2_ rocking, respectively. The two peaks at 509 and 403 cm^−1^ were due to Fe–O stretching in rGO/Fe_3_O_4_ NCs,^43^Fig. 3(d and e). The plant extract was used as reducing agent to facilitate reduction and formation of Fe_3_O_4_ NPs on the surface of rGO sheets without the need to use hazardous reducing agent such as hydrazine.

#### 3.1.3. FT-Raman analysis

**Fig. 4:**
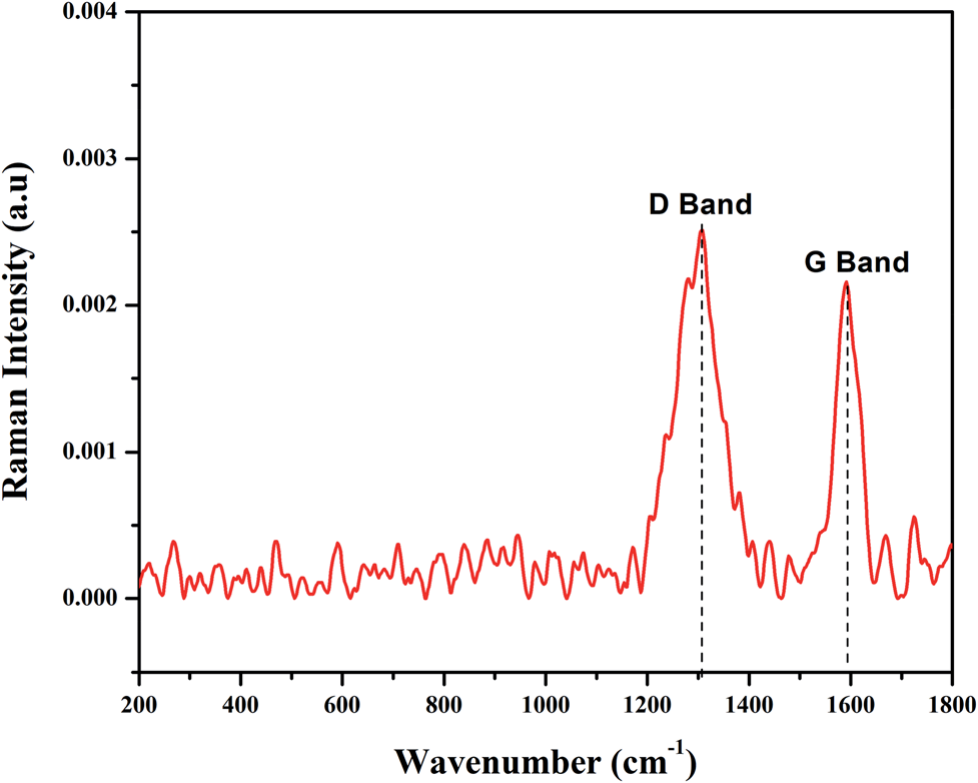
FT-Raman spectrum of rGO/Fe_3_O_4_ NCs.

FT-Raman spectroscopy is used to characterize carbon containing materials and to identify amorphous and crystalline carbon structures.^44^ GO was allowed to react with Fe^2+^/Fe^3+^ solutions in the presence of pod extract of *Dolichos lablab* L. as a reducing agent to produce rGO/Fe_3_O_4_ NCs. The alkaloid, phenol, flavonoid, amino acid, protein, terpenoid and saponin are the already identified constituents of *Dolichos lablab* L. that are responsible for reducing GO to rGO.^45^ The FT-Raman spectrum of rGO/Fe_3_O_4_ NCs is depicted in Fig. 4 and shows a clear formation of the nanocomposite. The FT-Raman shifts at 1306.92 cm^−1^ (D band) and 1591 cm^−1^ (G band) had ratio of *I*_D_/*I*_G_ = 1.19. The D band is assigned to the breathing mode of the *k*-point phonons with A_1g_ symmetry of disordered graphite structure, whereas the G band introduces the E_2g_ vibration mode between two sp^2^ carbon atoms.^46^ The three peaks at 269, 589, and 767 cm^−1^ were related to Fe–O vibration of E_g_ and A_1g_ modes in Fe_3_O_4_. Hence, this result also proved magnetite nanoparticles were decorated on surfaces of rGO sheets.^47^ Similarly, two peaks were absorbed at 1591 and 1307 cm^−1^ for magnetite–rGO composites synthesized by using hydrazine hydrate as a reducing agent.^7^ The G band of rGO (1601 cm^−1^, not shown here) is red shifted by 10 cm^−1^ to 1591 cm^−1^ in rGO/Fe_3_O_4_ NCs.

#### 3.1.4. Powder XRD analysis

**Fig. 5:**
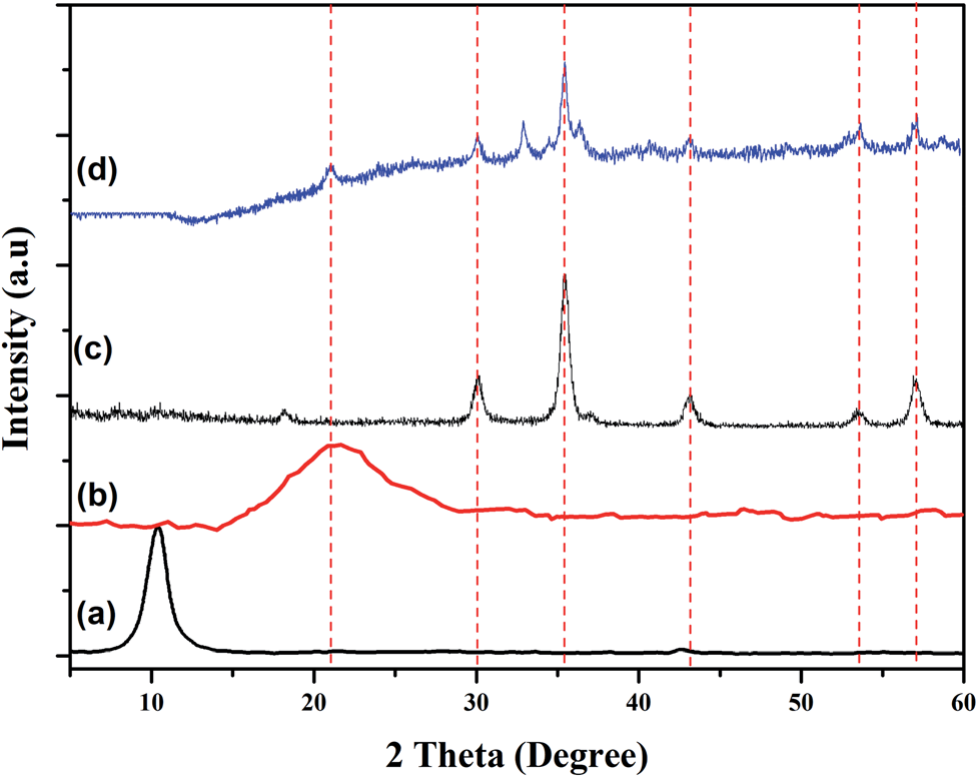
Powder XRD patterns of (a) GO, (b) rGO, (c) Fe_3_O_4_ NPs and (d) rGO/Fe_3_O_4_ NCs.

The phase structures of GO, rGO, Fe_3_O_4_ NPs and rGO/Fe_3_O_4_ NCs were characterized through powder XRD as indicated in Fig. 5. The XRD pattern of GO showed strong and intense peak at 2*θ* = 10° with a lattice reflection of (001), Fig. 5(a). The XRD analysis proved formation of rGO and displayed a broad and intense peak at 2*θ* = 21° with a lattice reflection of (002) in Fig. 5(b). Similar broad peak was observed in the angle between 15°–30° during synthesis of rGO using heating coconut shell.^48^ The XRD pattern of Fe_3_O_4_ NPs and rGO/Fe_3_O_4_ NCs in Fig. 5(c and d) displayed diffraction peak positions at 2*θ* values as 33.09°, 35.64°, 43.44°, 53.91°, 57.41° and 62.9° with diffraction lines matching to (220), (311), (400), (422), (511) and (440), respectively, are consistent with the standard XRD data for the face centered cubic (fcc) Fe_3_O_4_ structure (JCPDS card no. 19-0629). The peak at 2*θ* = 21° (002) which is seen in the XRD pattern of rGO/Fe_3_O_4_ NCs (Fig. 5(d)), confirmed Fe_3_O_4_ NPs were anchored on the surfaces of rGO sheets.^49^ The average crystallite size of the manufactured rGO/Fe_3_O_4_ NCs was calculated to be 38 nm using the well-known Scherrer equation (eqn (8)).8
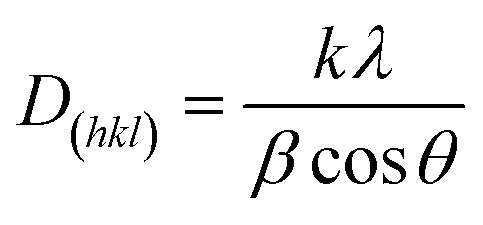
where, ‘*D*_(*hkl*)_’ is the average crystallite diameter (nm), ‘*k*’ is Scherrer constant (0.94), ‘*λ*’ is the X-ray wavelength, ‘*β*’ is the half width of XRD diffraction line, and ‘*θ*’ is the Bragg's angle in degrees.

#### 3.1.5. Morphology and structural analysis

**Fig. 6:**
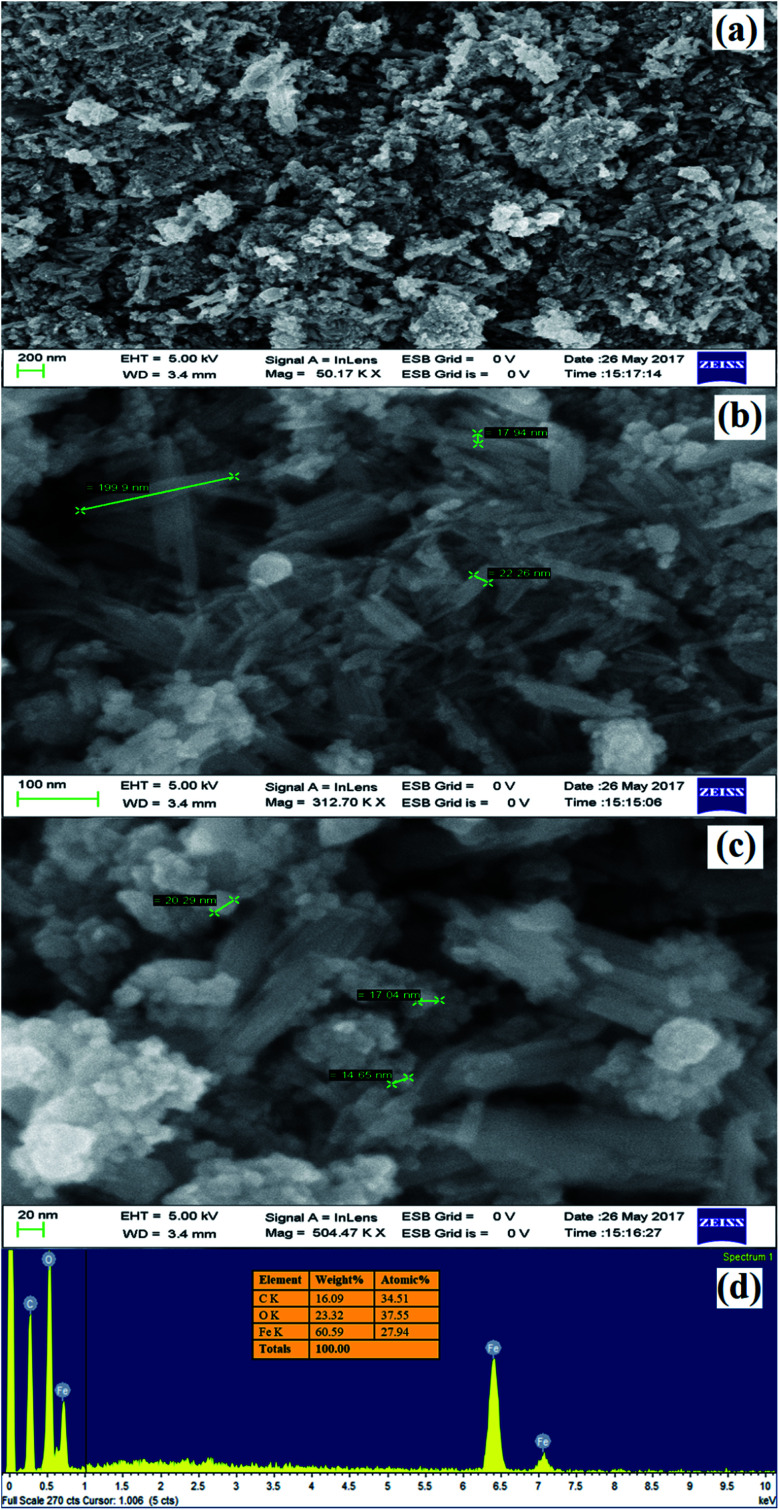
(a) to (c) FE-SEM images of rGO/Fe_3_O_4_ NCs at different magnifications and (d) EDX spectrum of rGO/Fe_3_O_4_ NCs.

**Fig. 7:**
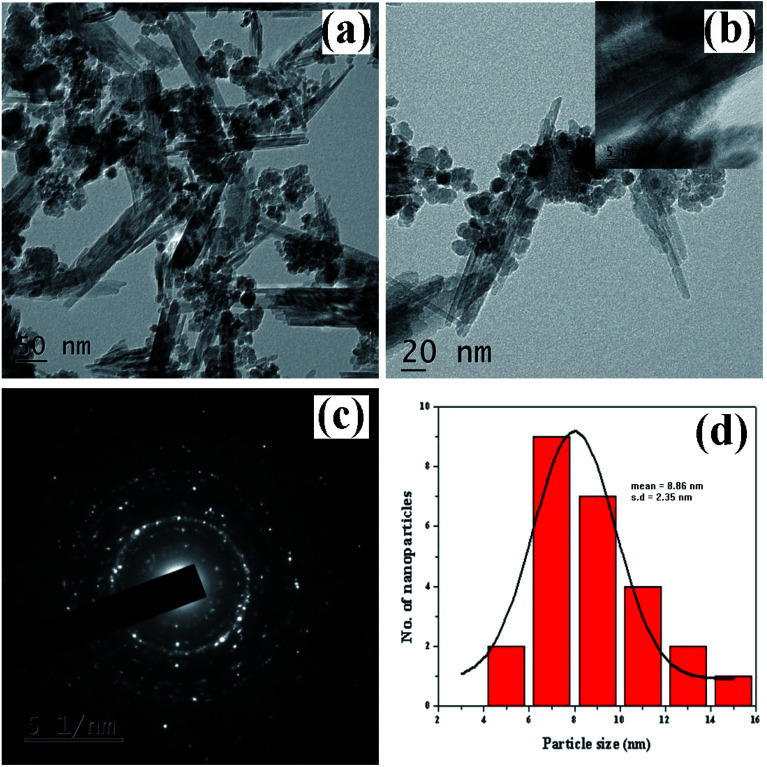
TEM images of *Dolichos lablab* L. mediated rGO/Fe_3_O_4_ NCs (a) 50 nm scale, (b) 20 nm scale, (c) SAED patterns of rGO/Fe_3_O_4_ NCs, and (d) size distribution histogram of rGO/Fe_3_O_4_ NCs.

The morphological looks and surface characteristics of rGO/Fe_3_O_4_ NCs were studied by using FESEM-EDX. Fig. 6(a–c) depicts FE-SEM images of Fe_3_O_4_ NPs that were consistently distributed on the surface of rGO sheets. The size of the Fe_3_O_4_ NPs from the image sources range from 9.75 to 14.85 nm. The EDX spectrum in Fig. 6(d) clearly identified the elemental existence of Fe, C and O with 27.94%, 34.51% and 37.55% by atomic mass, respectively. The TEM analysis was used to study the morphology and size of the synthesized rGO/Fe_3_O_4_ NCs. Fig. 7(a and b) clearly shows spherical shaped Fe_3_O_4_ NPs are homogeneously anchored at the surface of disorderly distributed rGO sheets. The SAED patterns of rGO/Fe_3_O_4_ NCs in Fig. 7(c) showed both polycrystalline nature of Fe_3_O_4_ and disorderly oriented nature of rGO sheets. The average particle size of Fe_3_O_4_ in rGO/Fe_3_O_4_ NCs was calculated to be 8.86 nm using (ImageJ) software, Fig. 7(d). There was small sign of aggregation of Fe_3_O_4_ NPs observed on the surface of rGO sheets. Hence, the large surface area to volume ratio of the NCs causes the rGO/Fe_3_O_4_ NCs to have an efficient adsorption property towards CV dye pollutant.

#### 3.1.6. VSM analysis

**Fig. 8:**
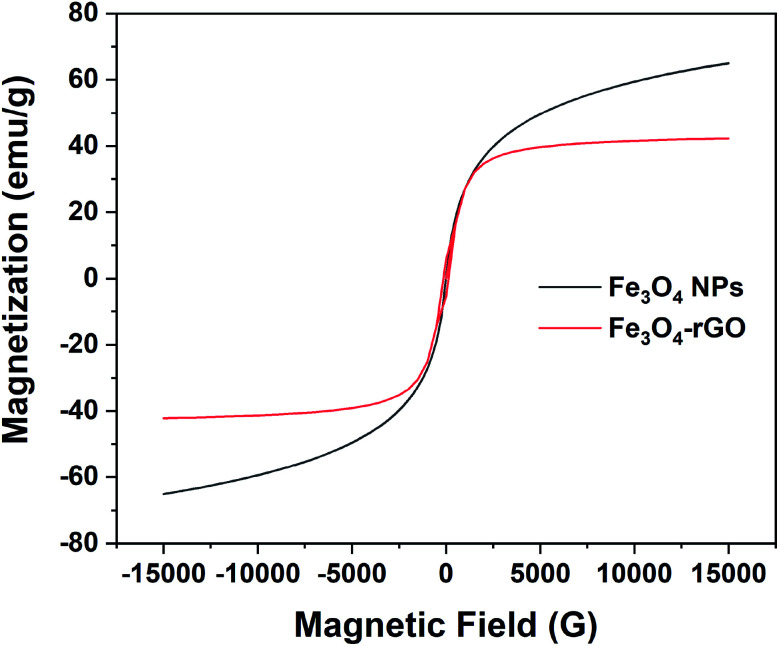
Room temperature magnetization curve of pure magnetite and rGO/Fe_3_O_4_ NCs.

The magnetic property of rGO/Fe_3_O_4_ NCs was measured by VSM at room temperature and confirmed its superparamagnetic property. Fig. 8 shows the hysteresis loop of rGO/Fe_3_O_4_ NCs with a magnetic saturation (*M*_s_) 42 emu g^−1^ which was lower than that of l-Met capped Fe_3_O_4_ NPs^50^ indicated Fe_3_O_4_ NPs were anchored on the surface of rGO sheets.

### 3.2. Adsorption study of CV dye

#### 3.2.1. Adsorption isotherms

**Fig. 9:**
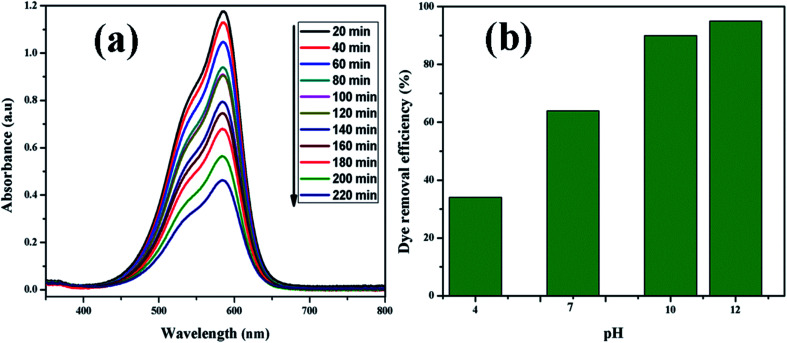
(a) UV-vis absorption spectrum of CV (20 mg L^−1^) adsorbed on rGO/Fe_3_O_4_ NCs at different time and (b) effect of pH on dye removal.

**Fig. 10:**
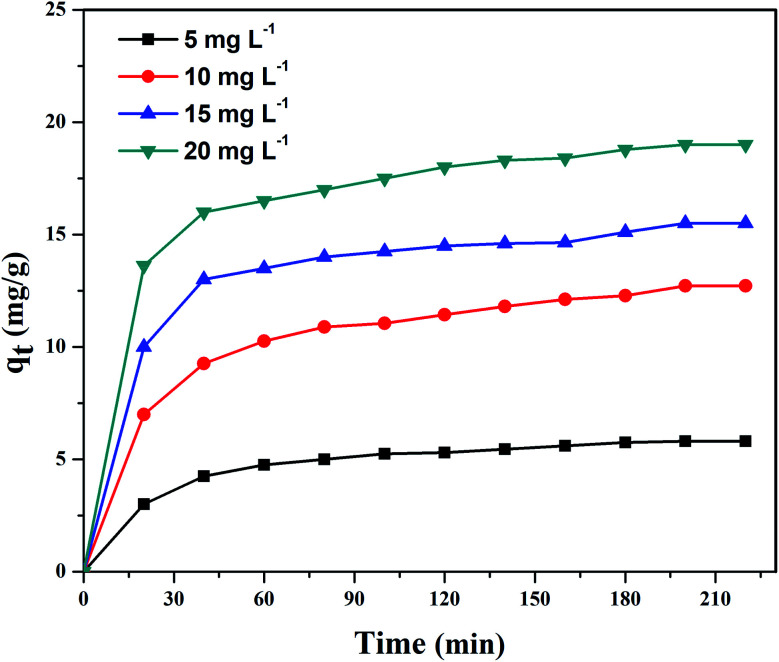
Adsorption of CV dye on rGO/Fe_3_O_4_ NCs at pH = 10 at different time.

**Fig. 11:**
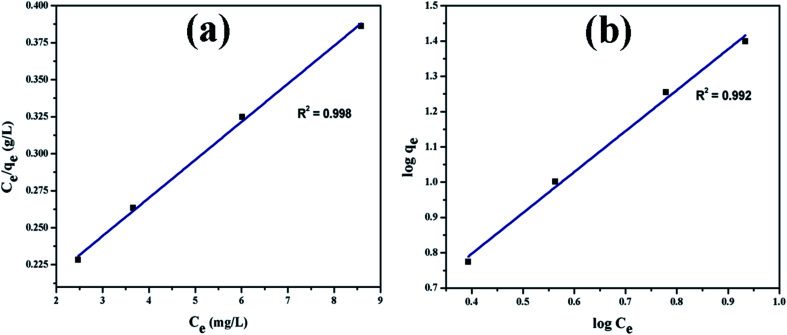
Adsorption isotherm plots of CV dye (a) Langmuir and (b) Freundlich isotherm.

Fig. 9(a) shows UV-vis absorption spectra of CV dye adsorbed on rGO/Fe_3_O_4_ NCs as a function of time, while Fig. 9(b) shows effect of pH on dye removal. The increase in the pH of dye solution (4, 7, 10, 12) resulted increase in the dye removal efficiency of the adsorbent by (34, 64, 90, 95%), respectively. The dye removal efficiency is lower at pH = 4 because the rGO/Fe_3_O_4_ NCs surface is positively charged. Similarly, Duman *et al.*^21^ have reported adsorption of CV dye onto magnetic OMWCNT–Fe_3_O_4_ NPs is lowest at pH = 2 (smaller than pH_PZC_). This is due to the electrostatic repulsion between positively charged adsorbent surfaces and positively charged CV dye. However, at the higher pH solution (pH = 12), the surface of the rGO/Fe_3_O_4_ NCs became negatively charged which resulted to electrostatic forces of attraction between CV dye molecules and the adsorbent that increased adsorption efficiency to 95%. Fig. 10, depicts adsorption isotherm of CV dye on rGO/Fe_3_O_4_ NCs using different initial CV dye concentration at pH = 10. Dye adsorption increased with increasing initial concentration of organic dye pollutant. Fig. 11, describes Langmuir and Freundlich adsorption isotherm models of CV on the surface of rGO/Fe_3_O_4_ NCs. The correlation coefficients (*r*^2^) and other parameters from the models are presented in Table 1. The adsorption isotherm better fitted to Langmuir (*r*^2^ = 0.998) than Freundlich (*r*^2^ = 0.992). Hence, the adsorption mechanism was monolayer, *i.e.*, physical intermolecular attraction between the adsorbent and adsorbate predominated. Similar results were reported for the adsorption isotherms of various pollutant-adsorbent systems.^51,52^ The monolayer adsorption maximum capacity (*q*_max_) of CV on rGO/Fe_3_O_4_ NCs was found to be 62 mg g^−1^. Hence, current study shows rGO/Fe_3_O_4_ NCs has better adsorption capacity than Fe_3_O_4_ NPs alone towards CV dye^53^ due to increased number of sites of functional groups in rGO/Fe_3_O_4_ NCs.

**Table 1:** Adsorption parameters of Langmuir and Freundlich equations and correlation coefficients for the adsorption of CV dye onto rGO/Fe_3_O_4_ NCs adsorbent at 25 °C

Adsorbent	Langmuir parameters			Freundlich parameters		
	*q*_max_ (mg g^−1^)	*K*_L_ (L mg^−1^)	*r* ^2^	*K*_F_ (mg g^−1^) (L mg^−1^)^1/*n*^	*n*	*r* ^2^
rGO/Fe_3_O_4_ NCs	62	0.150	0.9985	2.14	0.870	0.9925

#### 3.2.2. Adsorption kinetics

**Fig. 12:**
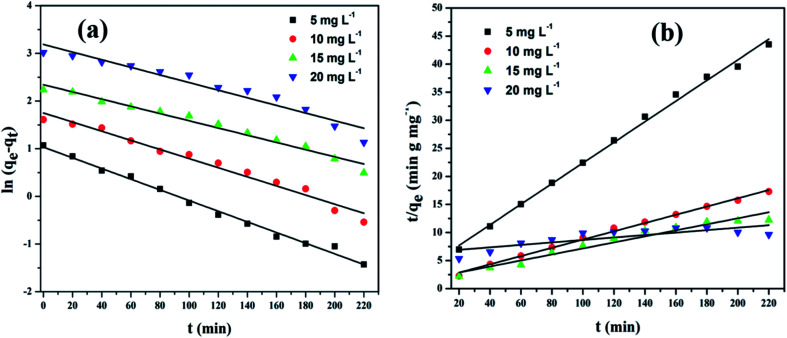
(a) Pseudo-first-order and (b) pseudo-second-order kinetic plots of CV on rGO/Fe_3_O_4_ NCs.

The linear plots of pseudo-first-order and pseudo-second-order kinetic models for adsorption of CV dye on rGO/Fe_3_O_4_ NCs are shown in Fig. 12. The kinetic model parameters and constants for the adsorption of CV dye on the surface of the adsorbent at room temperature are represented in Table 2. Adsorption of CV dye on surface of rGO/Fe_3_O_4_ NCs follows pseudo-second-order kinetics implies that the rate determining step is chemisorption.^54^ Similar results were reported for the adsorption kinetics of various pollutants onto activated carbon cloth.^55–57^ Therefore, adsorption was facilitated due to the interaction of cationic dye (CV) and rGO sheets through the oxygen functional groups and vacancy defects, by either electrostatic or π–π interactions.^58^ The maximum adsorption capacity of Fe_3_O_4_–rGO NCs synthesized by solvothermal method against methyl violet dye has been reported 196 mg g^−1^ by Cherukutty *et al.*^59^ Similarly, the maximum adsorption capacity of Fe_3_O_4_/GO hybrid to remove methylene blue dye from aqueous solution at 35 °C has been reported 96.05 mg g^−1^ by Liao *et al.*^60^ In addition, successful photocatalytic degradation of malachite green using Ag_3_PO_4_@MWCNTs@Cr : SrTiO_3_ composite from aqueous solution under sunlight or visible light irradiation has also been reported by Lin *et al.*^61^

**Table 2:** Kinetic parameters and correlation coefficients for the adsorption of CV dye onto rGO/Fe_3_O_4_ NCs at 25 °C

Initial CV concentration (mg L^−1^)	*q*_e_, exp (mg g^−1^)	Pseudo-first-order			Pseudo-second-order			
		*k*_1_ (min^−1^)	*q*_*t*_, cal (mg g^−1^)	*r* ^2^	*k*_2_ (g mg^−1^ min^−1^)	*q*_*t*_, cal (mg g^−1^)	*h* (mg g^−1^ min^−1^)	*r* ^2^
5	12.64	1.1 × 10^−2^	1.348	0.9923	8.4 × 10^−3^	12.12	1.342	0.9968
10	33.64	9.0 × 10^−3^	1.598	0.9737	3.9 × 10^−3^	32.52	4.413	0.9957
15	44.93	7.0 × 10^−3^	1.827	0.9745	1.6 × 10^−3^	42.26	3.230	0.9982
20	62.00	7.0 × 10^−3^	1.962	0.9346	7.3 × 10^−5^	61.97	0.2806	0.9989

### 3.3. Antifungal activity of rGO/Fe_3_O_4_ NCs

**Fig. 13:**
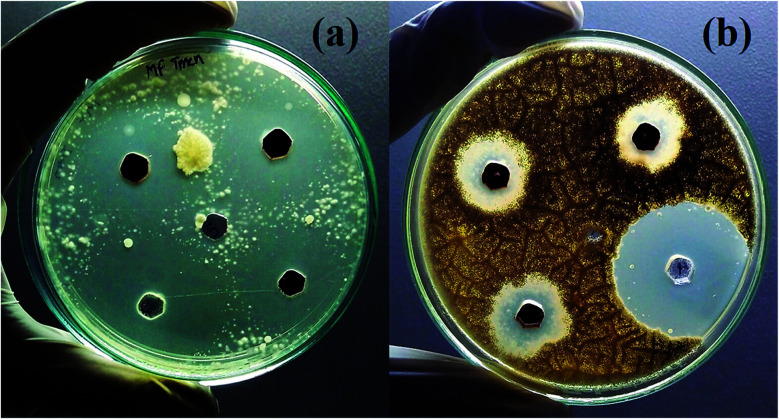
Antifungal activities of rGO/Fe_3_O_4_ NCs against (a) *Trichophyton mentagrophytes* and (b) *Candida albicans*.

**Fig. 14:**
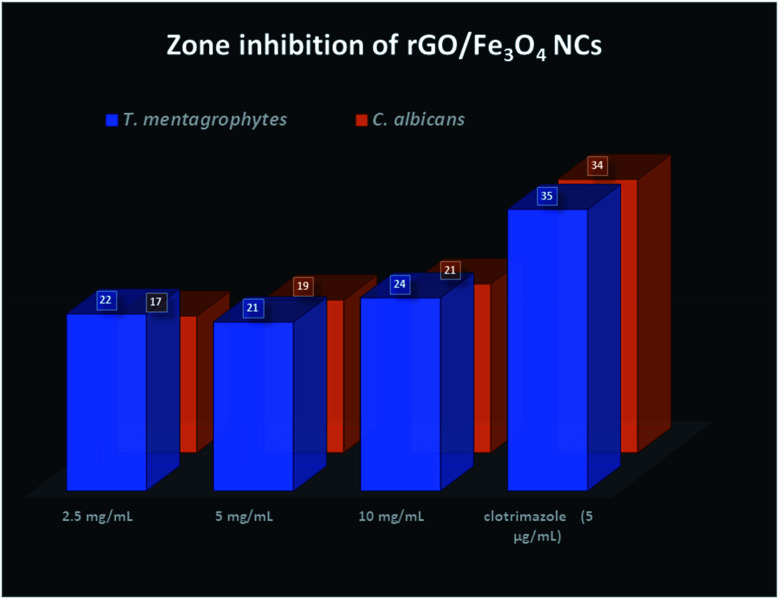
Zone inhibition of rGO/Fe_3_O_4_ NCs against pathogens.

Finally, the product rGO/Fe_3_O_4_ NCs showed antifungal activity against *Trichophyton mentagrophytes* and *Candida albicans* with maximum zone of inhibition of 24 mm and 21 mm, respectively. See both Fig. 13 and 14. Mohanta *et al.*^62^ reported biosynthesized AgNPs using aqueous leaf extract of *Erythrina suberosa* (Roxb.) showed antifungal activity against *Trichophyton mentagrophytes* with inhibition zone of 16 ± 0.8 mm, but no zone of inhibition was observed against *Candida albicans*. In addition, green synthesized ZnS NPs using *Phyllanthus niruri* plant extract and thorn-like ZnO NPs synthesized by sol–gel method both showed antifungal activity against *Candida albicans* with maximum inhibition zone of 32 mm and 20 ± 1.5 mm, respectively.^63,64^ Hence, the as-synthesized rGO/Fe_3_O_4_ NCs have showed better antifungal activity against the above studied fungus and therefore can be used as antimicrobial applications.

## 4. Conclusions

Eco-friendly, cost-effective and single-step green co-precipitation synthesis of magnetically separable rGO/Fe_3_O_4_ NCs using pod extract of *Dolichos lablab* L. as reducing agent was reported. The result of TEM analysis showed the average particle size of Fe_3_O_4_ NPs in the as-synthesized rGO/Fe_3_O_4_ NCs was 8.86 nm. The maximum CV dye removal efficiency of the adsorbent was calculated to be 95% at pH = 12. The adsorption mechanism well fitted to Langmuir than Freundlich isotherm and followed pseudo-second-order kinetics. *Dolichos lablab* L. mediated rGO/Fe_3_O_4_ NCs can be used as efficient removal of toxic dyes and metals from contaminated water and antifungal against microorganism pathogens.

## Conflicts of interest

There are no conflicts to declare.

## Supplementary Material
